# New Horizons for the Journal of Insect Science

**DOI:** 10.1093/jisesa/iez006

**Published:** 2019-02-08

**Authors:** Phyllis G Weintraub

**Affiliations:** 1 Editor-in-Chief, Journal of Insect Science; 2 Agricultural Research Organization, Gilat Research Center, Israel

As I look back on the first nearly 4 yr as editor-in-chief of the *Journal of Insect Science* (JIS), it seems like an excellent time to share updates and goals for 2019.

JIS now has an impact factor of 1.324, the highest in its history, and has the fastest submission to publication time of any ESA journal. Our dedicated subject editors work to maintain efficiency because we know that you, our authors and readers, want to see your work published with expediency and science made available as quickly as possible.

Table 1 presents data on our average speed from submission to first decision. Desk withdrawals and rejections can skew average times to look faster than they are for most papers, so you will see a separate line in which desk withdrawals/rejections (desk decision) are not included. In the table, you will see that the times from submission to decision have decreased each year since the Entomological Society of America (ESA) took over the journal. For papers that were sent into review, the average turnaround time to first decision was only 4.7 wk in 2018.

**Table 1.** Time from submission to first decision

Average no. of days from submission to first decision	2015	2016	2017	2018^*a*^
Desk decision included	29.6	27.3	24.9	20.6
Desk decision not included	44.7	42.7	41.6	33.3

Desk decision refers to papers withdrawn or rejected prior to peer review.

^*a*^As of 20 December 2018.

This coming year we have set several goals. One of the most important for me personally is to increase the percentage of women subject editors to reflect the percentage of women in ESA. Women have been under-represented for decades and all ESA journals will be striving to rectify that imbalance, including the *Journal of Insect Science*.

We will be starting two new areas of emphasis within the journal in 2019. The Entomological Society of America’s Medical, Urban, and Veterinary Entomology Section initiated a proposal to launch a subject section within JIS where Standardized Protocols will be published. MUVE wishes to encourage the creation of standard protocols that can save all entomologists time and reduce duplication of effort; as JIS is a fully open access publication, MUVE felt that JIS was an ideal place to publish such protocols and make them immediately and widely available. A special issue has been in preparation for the past few months that will be the flagship for this new area. Included with that issue will be instructions for authors that will ensure uniformity for reporting in all manuscripts, enabling the reader to know that they will consistently see complete and replicable protocols and results, and that they will not have to search through innumerable journals to find standardized protocols.

Another new section, Molecular Entomological Genetics, will be headed by Dr. Margaret Allen of the USDA, an entomologist with a robust background in molecular genetic insect science from Sanger sequencing to genetic manipulation. Dr. Allen will serve as the interface between the community of scientists wishing to publish molecular genetic research (genomics, transcriptomics and gene expression, gene characterizations, gene disruption, and other molecular topics) and the JIS subject editors. Dr. Allen will provide editorial guidance for journal policy on genetic data sharing, data archiving, and quality control. We aim to provide a focused venue for entomologically relevant molecular genetic research and ensure JIS articles are of the highest quality and credibility.

I strongly feel that JIS is continuing to fulfill and build upon Henry Hagedorn’s vision when he established JIS as an open access journal back in 2000. He wanted a platform for all insect scientists, from all regions the world, to have a dynamic and forward-looking journal to publish in. I think that by expanding the journal with these two new sections we will strengthen the importance and prestige of JIS, which will in turn allow us to better serve the entomological community.

